# Overlap between personality disorders and neurodevelopmental conditions

**DOI:** 10.1192/j.eurpsy.2025.268

**Published:** 2025-08-26

**Authors:** J. D. King

**Affiliations:** Division of Psychiatry, Imperial College London, London, United Kingdom

## Abstract

**Abstract:**

We all have a personality, whether or not we also have a mental health disorder, and for some people parts of our personality lead to problems with others, or in getting on in our lives. Indeed the same might be said of neurodevelopmental conditions, autism, intellectual disability, ADHD, of which we understand there to be a spectrum of degree, and which in unfavourable circumstances produces difficulty and dysfunction. The combination of personality and neurodevelopmental pathology is common, so too is misdiagnosis between them. This talk will describe the key theories to understand the overlap, tackle diagnostic uncertainty, and outlines the ways in which support can be offered for the combination of the two.

**Disclosure of Interest:**

None Declared

